# Gallbladder Diverticulum as a Rare Disease and a Therapeutic Challenge: A Case Report

**DOI:** 10.7759/cureus.61932

**Published:** 2024-06-08

**Authors:** Guangbin Chen, Yanguang Sha, Nana Guo, Yong He

**Affiliations:** 1 Department of Hepatobiliary Surgery, The Second People's Hospital of Wuhu, Wuhu Hospital Affiliated to East China Normal University, Wuhu, CHN; 2 Graduate School, Wannan Medical College, Wuhu, CHN; 3 Department of Pathology, The Second People's Hospital of Wuhu, Wuhu Hospital Affiliated to East China Normal University, Wuhu, CHN; 4 Department of Ultrasound Medicine, The Second People's Hospital of Wuhu, Wuhu Hospital Affiliated to East China Normal University, Wuhu, CHN

**Keywords:** atypical abdominal pain, surgical intervention, diagnostic challenges, laparoscopic cholecystectomy, gallbladder diverticulum

## Abstract

This case report details a rare instance of gallbladder diverticulum, highlighting the diagnostic and therapeutic challenges associated with this condition. Gallbladder diverticulum is an uncommon anomaly that often mimics the symptoms of more prevalent gallbladder diseases, making an accurate diagnosis challenging. The patient, a 55-year-old female, presented with atypical abdominal pain and was initially suspected to have chronic cholecystitis. Ultrasound examinations and subsequent enhanced computed tomography imaging revealed a gallbladder diverticulum without the presence of gallstones or polyps. Given the rarity of this condition and the potential for complications, a laparoscopic cholecystectomy was performed. The surgery was successful, and the patient's symptoms were completely resolved postoperatively, confirming the diagnosis. This report underscores the importance of considering gallbladder diverticulum in the differential diagnosis for atypical gallbladder symptoms and advocates for prompt surgical intervention to prevent complications. Our findings contribute to the limited literature on this rare condition and emphasize the need for awareness among clinicians to achieve optimal patient outcomes.

## Introduction

Gallbladder diverticulum, although rare, presents significant diagnostic and therapeutic challenges in clinical practice [1-4]. Characterized by the herniation of the gallbladder mucosa, this condition can manifest with symptoms similar to those of more common gallbladder diseases, complicating the diagnostic process. The pathogenesis of gallbladder diverticula involves a mix of congenital anomalies and acquired factors, with chronic inflammation and increased intraluminal pressure playing pivotal roles [1,4]. This case report explores the atypical clinical presentation of gallbladder diverticulum, emphasizing the complexities of its management and the critical role of timely surgical intervention. Through this discussion, we aim to enhance understanding and improve outcomes for patients afflicted with this unusual condition.

## Case presentation

**Figure 1:**
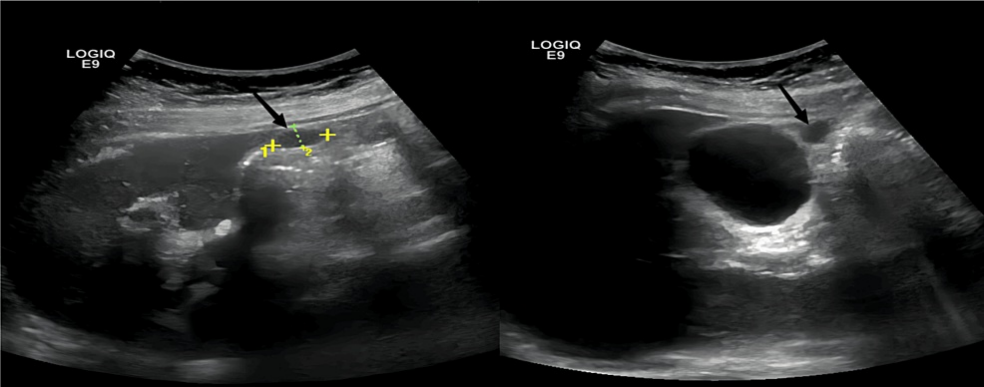
Outpatient abdominal ultrasound. The anterior wall of the gallbladder was localized outwardly expanded with an oval-like cystic cavity of about 17×9 mm in extent (black arrow), with clear boundaries and fair internal permeability, which was seen to be connected with the gallbladder cavity on dynamic scanning.

The patient, a 55-year-old female, exhibited no apparent triggers for the onset of recurrent episodes of right upper quadrant abdominal pain, which began 20 years ago, accompanied by back pain. The pain was mild and could resolve on its own. Each episode was managed with infusion therapy for infection, as the patient was unable to provide detailed treatment protocols. This alleviated the pain, and no other special treatments were performed. Over the past year, the patient reported that the pain had intensified. Consequently, the patient sought consultation at our hospital's hepatobiliary surgery outpatient clinic. An abdominal ultrasonography indicated chronic cholecystitis and a gallbladder diverticulum (Figure 1). 

The patient expressed a strong desire for surgery and was subsequently admitted to the hospital for a planned surgical intervention.

**Figure 2:**
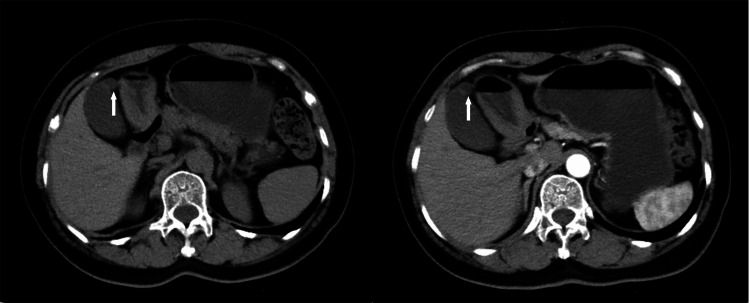
Preoperative CT of the upper abdomen. The gallbladder was full in appearance, no positive stone shadow was seen in the lumen, and a cystic pouch-like outgrowth was seen on the bottom wall (white arrow). CT: computed tomography

Throughout the course of the disease, the patient did not exhibit any significant jaundice of the skin or sclera, maintained a good appetite, and had normal bowel movements and sleep quality. Upon admission, further diagnostic tests were completed. The complete blood count showed a white blood cell count of 5.7×10^9^/L with 49.5% neutrophils. Liver function tests revealed alanine aminotransferase (ALT) at 22 U/L, aspartate aminotransferase (AST) at 19 U/L, alkaline phosphatase (ALP) at 52 U/L, gamma-glutamyl transferase (γ-GT) at 17 U/L, total bilirubin (TBiL) at 12.3 umol/L, direct bilirubin (DBiL) at 2.6 umol/L, and indirect bilirubin (IBiL) at 9.7 umol/L. Tumor markers were as follows: alpha-fetoprotein (AFP) at 2.48 ng/ml, carcinoembryonic antigen (CEA) at 2.3 ng/ml, cancer antigen 125 (CA125) at 6.8 U/ml, and cancer antigen 19-9 (CA19-9) at 33.1 U/ml. An upper abdominal computed tomography (CT) scan, both plain and enhanced, confirmed the presence of a gallbladder diverticulum (Figure 2).

**Figure 3:**
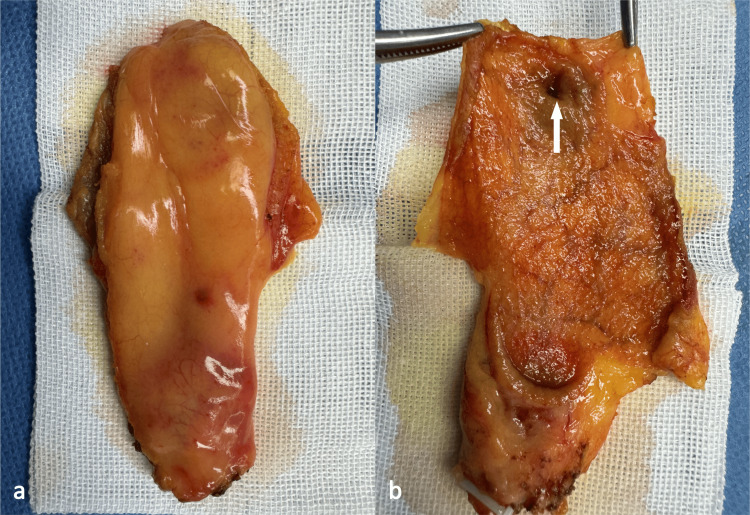
Gross specimen of the postoperative gallbladder. Outer view of the gallbladder (a), and inner view (b) shows a solitary cystic cavity expanding outward from the bottom of the gallbladder (white arrow), without calculi and breach of mucosa.

After ruling out any contraindications for surgery, the patient underwent a laparoscopic cholecystectomy (LC) under general anesthesia, which proceeded smoothly (Figure 3).

**Figure 4:**
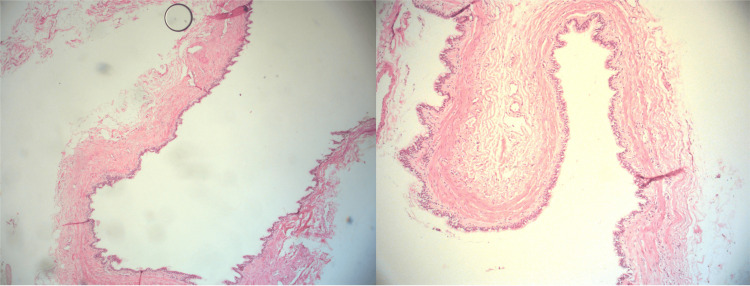
Postoperative pathology. Histologic appearance of the outpouching wall consisting of the mucosa, the muscularis propria, and the adventitia.

Postoperative pathology confirmed chronic cholecystitis and a gallbladder diverticulum (Figure 4).

Three months post-surgery, a follow-up call was made to the patient, who reported good recovery and no specific discomfort.

## Discussion

Gallbladder diverticulum is a rare and often underreported anatomical anomaly that presents significant diagnostic and therapeutic challenges [1,4-6]. This condition is characterized by the herniation of the gallbladder mucosa through the muscular layer, forming either a true (congenital) diverticulum, which includes all layers of the gallbladder wall, or a false (acquired) diverticulum, which lacks a complete wall structure [4]. The rarity of gallbladder diverticulum complicates its diagnosis and management, necessitating a thorough understanding of its pathogenesis, clinical presentation, and appropriate treatment strategies.

The pathogenesis of gallbladder diverticula is not fully understood but is believed to involve a combination of congenital and acquired factors. Congenital diverticula are likely due to embryological malformations, whereas acquired diverticula may result from increased intraluminal pressure, chronic inflammation, or ischemic conditions that weaken the gallbladder wall [4]. Chronic inflammation associated with gallstones has been suggested as a pivotal factor in the development of acquired gallbladder diverticula [3]. The diverticulum found in this study was a true diverticulum, as it was solitary, including three layers of the gallbladder wall, without calculi.

Clinically, patients with gallbladder diverticula may be asymptomatic or present with symptoms that mimic cholecystitis, such as right upper quadrant pain, nausea, and vomiting [1,4,7-9]. This overlap in symptoms can complicate the diagnostic process. In the case presented, the patient experienced recurrent episodes of right upper quadrant abdominal pain over a span of 20 years, which intensified in the past year, prompting further investigation.

Ultrasonography is the first-line imaging modality for diagnosing gallbladder diverticulum due to its accessibility and non-invasiveness [9,10]. However, its sensitivity can be limited, necessitating additional imaging techniques such as CT or magnetic resonance imaging (MRI) to confirm the diagnosis and rule out other pathologies. In this case, both ultrasonography and enhanced CT imaging were utilized to identify the gallbladder diverticulum, which was confirmed postoperatively.

The management of gallbladder diverticula depends on the clinical presentation and the presence of complications. Asymptomatic cases may be managed conservatively with regular follow-up [11]. However, symptomatic patients or those with complications such as recurrent cholecystitis or suspicion of malignancy typically require surgical intervention [6,9]. LC is the preferred surgical approach due to its minimally invasive nature and reduced recovery time compared to open surgery.

In this case, the patient presented with a small, solitary gallbladder diverticulum without gallstones or polyps but exhibited atypical symptoms. The decision to proceed with LC was based on the confirmed diagnosis of a true gallbladder diverticulum and the patient's persistent symptoms. The surgery was successful, and the patient's symptoms were completely resolved postoperatively, underscoring the importance of timely surgical intervention in managing this rare condition.

## Conclusions

Managing gallbladder diverticulum requires vigilant detection and a collaborative approach among ultrasound experts, imaging specialists, pathologists, and clinicians. This case highlights the crucial role of timely surgical intervention in preventing complications and ensuring positive outcomes. A multidisciplinary strategy is essential to effectively address the complexities associated with this unusual gallbladder pathology. Enhanced awareness and understanding among clinicians can lead to better diagnostic accuracy and optimal patient care.
